# Attitudes of Arab Family Caregivers in Israel Toward Robot-Delivered Care For ADL-Dependent (Activities of Daily Living-Dependent) Older People: Identifying Ethical and Social Issues

**DOI:** 10.1007/s11673-024-10414-3

**Published:** 2025-04-09

**Authors:** Hanan AboJabel, Fareeda Abo-Rass

**Affiliations:** 1 The Paul Baerwald School of Social Work and Social Welfare, The Hebrew University of Jerusalem, Jerusalem, Israel; 2 Takemi Program in International Health, Harvard T.H. Chan School of Public Health, Boston, MA USA

**Keywords:** Family caregivers, Elder care obligation, Loneliness, Robot, Technological literacy, Activities of daily living (ADL)

## Abstract

Older people are often cared for by family caregivers who may experience a variety of challenges. Robots may be helpful. Understanding the attitudes of family caregivers in this context is essential as they are a major factor in robot use. To date, most studies on family caregivers ‘ attitudes toward robot assistance in elder care have been conducted in East Asian or Western societies, but as such perceptions are affected by culture, in the present study, we investigated the attitudes of Arab family caregivers in Israel. Semi-structured interviews were conducted with twenty Arab family caregivers who provide care to ADL (activities of daily living)-dependent older people. All participants were Muslim and adult children of a person requiring care. The majority (75 per cent) were women, and the average age was fifty-one. Analysis of the interviews revealed two main themes: (1) Attitudes: Most participants strongly objected to the assistance of a robot in caring for older people, and perceived it as a violation of family values; and (2) Reasons for attitudes: Participants preferred human care over robot care due to concerns previously documented among other populations regarding system malfunctions, the risk of loneliness, and lack of technological literacy. We also identified a unique factor: the family ‘s moral obligation to care for older people. The absolute opposition of family members to the use of a robot in the care of older people requires the development of intervention programmes to increase technological literacy among family caregivers and reduce negative attitudes. These programmes need to address the opportunities and risks associated with the use of robots, and how these risks can be avoided.

## Introduction

Life expectancy is increasing rapidly worldwide, and aging is often accompanied by morbidity and disability that lead to reliance on others for activities of daily living (ADLs), including mobility, bathing, eating, toileting, and dressing (Cwirlej-Sozanska et al. 2018; Rong et al. 2019; Zhang et al. 2016). Family members, especially first-degree relatives such as adult children, are the primary source of care for older people, particularly in traditional collectivist societies, which are characterized by a high level of commitment to caring for older people (Carr and Utz 2020; Hammad et al. 2024; Hanssen and Tran 2019).

Caring for older people is associated with several negative consequences, such as caregiver burden, depression, and anxiety symptoms and a decline in family caregivers‘ quality of life and quality of care (Gagliardi et al. 2022; Kim 2017; Zhong et al. 2020). Technology in general, and robots in particular, may reduce such negative outcomes and various challenges related to caregiving (Lindeman et al. 2020; Shishehgar et al. 2018; Wang et al. 2017). For instance, robots may improve the independence and safety of older people at home by helping them with housework, reminding them to take medication, or providing companionship), and by supervising the activities of old people, they may reduce the burden of family caregivers, including care time and stress (Kim et al. 2022; Suzumura et al. 2020).

### Delivery of Care for Older People via Robots

The International Organization for Standardization (2012) describes robots as machines that perform specific tasks autonomously to a certain extent and without human intervention. Indeed, there is a wide variety of robots designed for perform different purposes, which can be classified into nine main areas: (1) a companion robot offers the opportunity for interaction and companionship; (2) a telepresence robot can facilitate remote communication with others and can also be used for surveillance; (3) a manipulator service robot is designed to carry and deliver objects, such as water, on demand; (4) a rehabilitation robot provides physical assistance, such as walking aids and walking chairs; (5) a health-monitoring robot monitors various health parameters such as heart rate and blood pressure; (6) a reminder robot reminds the individual to perform several actions such as taking medications or attending appointments; (7) a domestic robot helps carry out daily household chores; (8) an entertainment robot provides different forms of entertainment; and (9) a prevention robot detects or prevents falls (Shishehgar et al. 2018).

Some of these robot types are not yet available and are in the process of development and evaluation, such as ROBEAR, designed to perform tasks such as safely lifting elderly people from a bed to a wheelchair or helping them stand if they need support (Faisal et al. 2024). Some are already in the market, such as Care-O-bot, designed as a domestic robot assistant; the seal-like robot Paro, which functions as a social companion; and Cutii, a telepresence robot specifically designed for older individuals (Aguiar Noury et al. 2021; Fischinger et al. 2016). Studies show that providing care to older people with the assistance of robots may help them maintain their independence and autonomy in performing daily activities, ensure their safety, and enable them to communicate with others more easily (Bedaf et al. 2016; Halicka and Surel 2022; Löbe and AboJabel 2022; Samuel et al. 2023; Sumner et al. 2021). In addition, interactions with robots such as companion robots have positive effects on physiological (e.g., blood pressure) and psychological (e.g., loneliness) aspects (Robinson et al. 2014, 2015). Thus, robots may be effective in promoting aging in place—that is, enabling old people to age in their own home and community for as long as possible (Li and Liu 2022; Sumner et al. 2021).

### Family Caregiver Attitudes

In general, studies that have been conducted in East Asian societies (e.g., Japan), as well as in Western societies (e.g., the United States and European countries), on attitudes of family caregivers have shown that family caregivers are open to and welcome the delivery of care to older people via the assistance of a robot (Kodate et al. 2022; Mast et al. 2010; Suwa et al. 2020; Wang et al. 2017; Yuan et al. 2022). Robots can assist family caregivers by reducing burden, stress, and care-related concerns; increasing peace of mind; and giving them a break from caregiving (Mast et al. 2010; Wang et al. 2017).

For instance, an experiment conducted in China among twenty-seven family caregivers of older people with cognitive decline who used a robot for six weeks was designed to provide the following: companionship for the older person by talking to them as well as responding behaviourally to any sensory interaction (for example, when the older person holds the robot’s hand, the robot responds), various entertainment activities (for example, reading stories or songs), reminders (such as taking medication), and monitoring the older person’s activity. It was found that the study group (*n* = 19), compared to the control group (*n* = 8), experienced a lower level of role strain as a result of using a robot (Kim et al. 2022).

### Ethical Concerns

Despite the expected benefits of providing care to older people via a robot, both for the older people and for the family caregivers, a variety of moral and ethical issues have been described in the literature that may constitute a main barrier to use among various users (Ide et al. 2024; Martínez-López et al. 2023). One worrisome ethical issue that has been widely studied in the literature is older people’s increased risk of loneliness as well as isolation due to the replacement of human care by machines (AboJabel et al. 2024; AboJabel and Ayalon 2023; Noori et al. 2019; Sorell and Draper 2014). In addition, relying on robots instead of human beings puts older people at risk of injury and threatens their security due to potential malfunctions in the robot’s operation (Badr and Dankar 2022; Fasoli et al. 2023; Papadopoulos et al. 2020). Another common ethical issue is privacy-related; for instance, using a telepresence robot for surveillance of older people means that their privacy may be invaded by family members. In addition, various components are often incorporated into robots to enable them to perform their tasks efficiently such as sensors, microphones, cameras, and speakers. These components can collect a vast amount of personal and sensitive data on their health and daily schedules, which may be at risk of misuse, theft, and unauthorized distribution to manipulate and defraud the older person (Blake 2020; Lutz and Tamó-Larrieux 2020; Martínez-López et al. 2023; Niemelä et al. 2017; Sharkey and Sharkey 2012; Torresen et al. 2018).

A family ‘s moral obligation to care for their older family members may also constitute a moral dilemma, which has not been studied to date. This dilemma stems from the notion that the care of older people should not be transferred to technological entities, as doing so is inconsistent with prevailing family values regarding caring for older people via physical visits that include instrumental and emotional assistance. In addition, technology makes it easier for family caregivers to care for their relatives remotely, without being physically present. Technology can thus serve as a way of easing the conscience of family members and assist them in not fulfilling their filial obligation toward their parents (AboJabel and Ayalon 2023).

### Cultural Differences

Although studies focusing on cultural differences in perceptions of robot use in the care of older people are extremely limited, such studies have indeed shown that these perceptions may differ across cultural groups (AboJabel and Ayalon 2023). For instance, studies comparing Japan and European countries (Ireland and Finland) revealed that in Japan there were more positive attitudes toward the use of robots in caring for older people than in Ireland and Finland. The researchers explained that this finding may have resulted from Japanese society’s greater familiarity with robots, as well as their more advanced development of robots compared to that of other countries. They also attributed this finding to the policy of caring for older people, which supports and emphasizes robotics. Finally, they believed the differences found in their study were related to cultural differences, but did not explain exactly how culture was related to the differences in the perceptions of robots (Coco et al. 2018; Suwa et al. 2020).

A recent study conducted among a representative sample of Jews in Israel—a country ranked somewhere between collectivism and individualism—found that people with a highly collective orientation would likely report higher negative attitudes toward robotic assistance in the care of older people than would people with a highly individualistic orientation. Such attitudes included the perception of possible negative consequences: namely, that using a robot might endanger older people’s lives, might negatively affect relationships with older people’s family members, and might cause older people to experience a high level of loneliness. In addition, they were likely to report more negative attitudes toward family caregivers who used a robot and, therefore, presumably did not take great enough responsibility for their loved ones (AboJabel and Ayalon 2023).

According to Hofstede (Hofstede Insights 2020), people from collectivist societies tend to have a high level of uncertainty—that is, the level at which a person feels threatened by uncertain and incomprehensible situations—and therefore tend to adopt fewer technological innovations, including the use of robots to assist in the care of older people (AboJabel and Ayalon 2023). In addition, other studies have shown that in collectivist societies (e.g., Islamic or East Asian countries) the role of caregivers is culturally embedded (Hammad et al. 2024; Hanssen and Tran 2019; Pharr et al. 2014). These aspects might explain why people from collectivist societies tend to report negative attitudes about using robots in the care of older people.

### The Current Study

Despite the importance of existing studies on family caregiver attitudes toward providing care for older people via a robot, most have been conducted only in East Asian or Western societies (Kim et al. 2022; Mast et al. 2010; Suwa et al. 2020; Yuan et al. 2022; Wang et al. 2017). To the best of our knowledge, this topic has not been studied among Middle Eastern cultures. Therefore, the current study explores the attitudes of Arab family caregivers in Israel toward robot-assisted care for ADL-dependent older people, with a focus on understanding participants’ social and ethical perceptions.

Arabs constitute the largest minority group in Israel (about one-fifth of the population) and are composed mostly of Muslims (85 per cent), with a smaller number of Arab Christians, in addition to Druze, Circassians, and others (Israel Bureau of Statistics 2022). Arabs and Jews in Israel have separate religions, cultures, and languages. The two groups are unequal not only in terms of education and income, but also in terms of health (Dallasheh and Zubeidat 2023; Saabneh 2016). For example, according to estimates from the Central Bureau of Statistics in Israel (2022), between 2019 and 2021, 8.5 per cent of older Jews compared to 30.1 per cent of older Arabs had significant difficulty with personal care (i.e., dressing and bathing). Additionally, 19.3 per cent of older Jews compared to 51.9 per cent of older Arabs experienced great difficulty in performing household activities (Myers-JDC-Brookdale Institute 2023). Furthermore, compared to Jews, older Arabs are at a higher risk of developing chronic diseases (such as diabetes and cancer) and neurological diseases (such as dementia), as well as poor mental health, including depression symptoms (AboJabel et al. 2015; Chernichovsky et al. 2017a, 2017b; Dwolatzky et al. 2017; Saabneh et al. 2016; Werner et al. 2015). Increased health risks among older Arabs may be related to low socioeconomic status, lack of knowledge and awareness regarding mental and physical illnesses in aging, as well as unhealthy lifestyles (Chernichovsky et al. 2017a, 2017b; Dwolatzky et al. 2017; Werner et al. 2015). Despite the high rates of morbidity and disability among older Arabs, and despite the cultural and social changes that Arab society in Israel has been undergoing, this population is characterized by strong family values, a traditional orientation, and a strong familial commitment to providing care to older people (Ayalon 2015, 2018).

Like their Jewish counterparts, Arab older people have access to medical services provided in community clinics and hospitals. In addition, they have access to nursing homes and community services (e.g., day centres). The responsibility and operation of these services is divided between health maintenance organizations (HMOs), the Ministry of Welfare, and the Ministry of Health. Despite the availability of these services, the Arab population in general, and Arab older people in particular, are less likely to consume them due to various reasons. Compared to the Jewish population, the majority of the Arab population lives in peripheral areas, which are characterized by a lack of services and the low quality of those that are available. Furthermore, several services are not culturally sensitive to the needs of the Arab population (such as language barriers due to poor command of Hebrew). In addition, the lower digital literacy among the Arab population reduces the use of online services, and stigma and negative cultural attitudes towards welfare services are common (Chernichovsky et al. 2017b; Laron and Isaaq 2020; Naim et al. 2017; Shibli et al. 2021; Vitman-Schorr and Khalaila 2022; Werner and Tur-Sinai 2024). Thus, it is not surprising that Arab caregivers report feelings of burden and ambivalent feelings toward care more than do Jewish family caregivers (AboJabel et al. 2021).

## Methodology

Qualitative research was conducted via semi-structured interviews with Arab family caregivers of ADL-dependent older people. Semi-structured interviews allow participants to express their perceptions and experiences, allowing for a deep understanding of the study topic (Kakilla 2021).

### Participants

The study included twenty Arab family caregivers of ADL-dependent older people. The inclusion criteria were: (a) being the main informal caregiver (i.e., providing ongoing emotional and instrumental care and assistance without pay) for an older person who has difficulty performing one or more of these activities independently: bathing, dressing, toileting, mobility, and feeding, for at least six consecutive months; and b) being an Arabic speaker. The exclusion criterion was being a family caregiver who provides care for older people with cognitive decline or dementia, as dementia impairs an older person’s ability to make decisions. Dementia may therefore create unique ethical challenges in caring for such individuals and becomes an even more complex issue in the context of technology use (Hine et al. 2022).

### Procedure and Sampling

Participants were recruited from villages and cities in northern Israel, using three main sources: social workers working with this population at local welfare offices, day centres for older people, and through personal acquaintance. The interviews were conducted until saturation of new information was reached (Hennink et al. 2017). The interviews were held in August and September 2023, face-to-face at the participant’s home (*n* = 12) or over the phone (*n* = 8), according to participants’ preference. Prior to the interviews, participants signed an informed consent form. One of the researchers (HAJ) carried out the interviews in the native language of the participants (i.e., Arabic). The average interview duration was about twenty minutes.

### Interview Guide

In accordance with an interview methodology recommended for qualitative research (Kallio et al. 2016), a semi-structured interview guide was developed (see appendix 1). Because the research issue is an innovative one, with which some participants had no familiarity or prior exposure, we used a hypothetical vignette to illustrate the delivery of care to older people by a robot. The vignette was developed by AboJabel and Ayalon (2023) and included a description of three children using a robot to help provide care for their mother Dahlia. Dahlia was described as a seventy-year-old widow with three children, living alone in a large apartment, and having high blood pressure and diabetes. The robot in the vignette was designed to provide the following functions: assistance with daily household tasks, entertainment, reminder to take medications, two-way communication, and the carrying/delivering of objects on demand. Although currently a robot that can perform these tasks independently and autonomously is not available for commercial use, it is common in research to present such vignettes with the aim of exploring and understanding attitudes in the research context (AboJabel and Ayalon 2023; Hoppe et al. 2023; Schönmann et al. 2023). After reading the vignette, the researcher addressed with the participants the consequences of using a robot for Dahlia and her children and also talked about their readiness to use such a robot in caring for their relatives and how it could help them face challenges associated with care.

### Data Analysis

Interview analysis was conducted using a five-step thematic analysis approach (Terry et al. 2017). In the first step, the researchers read the transcripts several times to familiarize themselves with the data. In the second step, a hybrid deductive and inductive process was used for initial coding; that is, coding was done deductively based on the objectives and the study’s research question, and inductively by identifying new codes and subcodes. In the third stage, the main characteristics that made up the categories were grouped into potential themes and sub-themes, which were discussed by the two researchers. In the fourth step, the researchers identified and selected names for the themes. The themes were re-examined to pinpoint specific characteristics for each theme that emerged from the analysis, creating a definition and a clear name for each theme. The final step was to compile appropriate citations to illustrate the theme. The interviews and interview analysis, including the coding process, were conducted in Arabic. Specific quotes were translated from Arabic into English in a back-and-forth process by both researchers independently.

### Ethical Considerations

The study’s protocol was approved by the Ethics Committee of the School of Social Work of the Hebrew University of Jerusalem, with the approval number 03082023.

## Findings

### Characteristics of the Participants

A total of twenty Arab caregivers of ADL-dependent older people were included in the study. All participants were adult children, Muslims, and lived in northern Israel. The majority were female (75 per cent) and married (65 per cent). Their mean age was fifty-one (SD = 6.87; range = 36–62), and they had an average of twelve years of education (SD = 2.73; range = 7–18). Participants had been involved in their relative’s care for an average of 3.75 years (SD = 1.54, range = 1–9) and provided an average of twenty-seven (SD = 14.7, range = 7–60) hours of care per week. Finally, 80 per cent of family caregivers reported having an average income, 15 per cent reported having a below-average income, and only 5 per cent reported having an above-average income. Regarding the characteristics of the care recipients, most were women (60 per cent), and their average age was 77.4 years (SD = 5.83; range = 70–87). The participants’ characteristics are summarized in Table 1.

**Table 1:** Participants’ characteristics (*n* = 20)

*Family caregivers’ characteristics*	
Mean age (SD, range)	51 (6.87, 36–62)
Gender (%)	
Female Male	75 25
Mean years of education (SD, range)	12 (2.73, 7–18)
Marital status (%)	
Married Unmarried	65 35
Religion (%)	
Muslim	100
Type of relative (%)	
Adult children (son/daughter)	100
Economic situation	
Below-average income An average income Above-average income	15 80 5
***Caregiving characteristics***	
Mean years of caring (SD, range)	3.75 (1.54, 1–9)
Mean hours of weekly caring (SD, range)	27 (14.7, 7–60)
***Care recipients’ characteristics***	
Mean age (SD, range)	77.4 (5.83, 70–87)
Gender (%)	
Female Male	60 40

*SD* standard deviation

### Themes

Analysis of the interviews revealed two main themes: (1) Attitudes: Most participants strongly objected to the assistance of a robot in the care of older people and perceived such assistance as a violation of family values, and (2) Reasons for attitudes: concerns about system malfunctions, risk of loneliness, lack of technological literacy, and filial/moral obligation to care for older people.

#### Theme 1. Attitudes Regarding the Use of Robot for the Care of Older People

In general, the study participants were unanimously opposed to using robots to care for their loved ones, as they believed human care to be necessary for older people in all care tasks and at all times. Therefore, they considered the division of care among family members to be the most effective way to care for older people. If family members were unable to provide care themselves, they suggested the use of formal services including institutional (such as nursing home) and community services (such as long-term care benefit) to ensure the presence of the “human factor.”

#### Sub-theme 1.1 Opposition of family caregivers to robots in helping to care for older people

Most participants (18/20) had a negative attitude toward robots and were strongly opposed to caring for older people via the use of a robot, even for a short time (i.e., a few hours). This opposition persisted regardless of the specific care task meant to be provided by the robot. Moreover, family caregivers grew irritated by the mere mention of the topic and did not wish to address it. They felt that the “human factor” was crucial in the care of their loved ones, especially the care provided by family members.

I am not at all ready to use a robot to take care of my mother. We are not in this situation... and I don’t think we will ever arrive at this situation! I’m referring to the idea of a robot taking care of the elderly... or taking care of my mother specifically. I haven’t considered it at all (P1, a daughter).

Why would a robot help my mother? A piece of metal doesn’t take care of one’s mother... I’m not interested in the subject at all, and I don’t want to think about a machine taking care of a person’s mother (P18, a daughter).

I am not ready to use a robot in the care of my mother. No way!!! Not even for a few hours a day (P11, a daughter).

Only two participants were willing to discuss this issue, and they favoured “hybrid-care” (i.e., combining human and robot care). They believed that integrating a robot into the care of older people for a few hours a day could lead, especially in tasks that require physical labour (such as household chores), to a reduction in the burden of care and other conflicts that may arise in this context, such as conflicts at work or in the family.

Yes, a robot can help Dahlia if her children are busy and cannot be present on a certain day... but they must still go to her. The robot can do the physical work... at the same time the children must continue to come to their mother. The children must be present, for example, they must feed her. Even if the robot prepares the food, they must be present when the food is prepared and eat with Dahlia. The robot can help the adult child do all kinds of work, but it is not acceptable not to go to her or visit her. The robot cannot be a substitute for the children (P12, a daughter).

In addition, the participants who were willing to consider “hybrid-care” saw the robot as a tool that could increase the older person’s independence, thus potentially improving the relationship between the older person and the person’s family members.

Surely this [the robot] strengthens her [Dahlia’s] independence. Instead of her having to wait for her children to help, she does not have to depend on anyone, and the robot can help her. This will improve her relationships with the children. They will not be under pressure, knowing that their mother needs help. After all, the children work, and they also have children [….] (P15, a son).

#### Sub-theme 1.2 Division of care among family members

Participants stated that caring for an older person should be done first and foremost by family members, who understand the older person’s needs and can care for them with affection. However, given that caring for an older person can be a difficult and complex task, cooperation by all family members is required. Participants therefore concluded that the most effective way to provide care for older people was by dividing care responsibilities between family members, for example, by working in shifts.

In my opinion, the children should have taken care of Dahlia by themselves, without the use of a robot. Especially since there are three of them, and not only one. They could work in shifts and not put all the burden on one [...] The older woman needs her children (P7, a daughter).

I believe the children should work in shifts; they should share the caring between them (P4, a daughter).

#### Sub-theme 1.3 Seeking formal services for the care of older people

Participants indicated that in the event that family members are unable to care for their older loved ones, due to matters such as work or having to take care of other family members (e.g., children), they should seek formal services for their older relative, rather than using a robot. Although family caregivers would be less involved in the care of their relative (i.e., if they used formal services instead), the care would still involve the “human factor.” Participants suggested, for example, employing paid caregivers for several hours a week through long-term care benefit granted to ADL-dependent older people by Israel’s National Insurance Institute (NII):

Yes, I think it shouldn’t be done [using a robot]. A paid worker should be brought in to help her [Dahlia], if the children cannot do so. The children can apply to receive a caregiver from the NII (P3, a daughter).

Participants also suggested employing a full-time worker, some of whose salary can be financed by the NII:

It is also possible to bring in a foreign worker, who will take care of her [Dahlia] 24/7. They [Dahlia’s children] can pay part of the salary through the State’s nursing allowance, and the children will pay the other part (P19, a son).

In addition, they said that moving Dahlia to a nursing home would be better than providing robot care, because in a nursing home, human care and social relationships are present:

If the children do not have time to take care of their parents, it is better to move the parents to a nursing home instead of bringing in a robot. At least in a nursing home the parent will have connections with other people (P16, a daughter).

She [Dahila] can go into a nursing home; it’s better than a robot. In a nursing home she can talk to people; they do all kinds of activities in a nursing home, she won’t be left alone (P3, a daughter).

#### Theme 2. Reasons for Attitudes

We identified four key factors that caused family caregivers to express a preference for human care over robot care, including a) filial/moral obligation to care for older people, b) participants’ lack of technological literacy, c) occurrence of system malfunctions, and d) older people’s loneliness.

#### Sub-theme 2.1 Filial and moral obligation to care for older people

The main reason described by participants to avoid using a robot in the care of older people was a feeling of moral obligation and responsibility to older people, especially one’s parents. For example, participants said that their parents raised them and dedicated their whole lives to them. When they become older, therefore, they should be cared for accordingly:

[...] I think it is very important to take care of our parents ourselves. Because they raised us, they took care of us when we were small [...]. I want to offer some advice, and that is that everyone who has parents should help them themselves (P3, a daughter).

In addition, participants described the use of robots in the care of old people as a means of abandoning older people, and as a way to shirk their responsibilities:

There is no replacement for the children, who alone can give emotional warmth to their parents. Those who use robots to take care of their parents are trying to get rid of their parents. They want to throw their parents away. If the children are not physically with the old person, then it is not worthwhile, it won’t help. As important as technology is, there is nothing like having the children around themselves (P13, a son).

Furthermore, participants described the robots as a tool that removes the burden from the caregiving family members and makes it easier for them (i.e., the use of robots was seen as selfish):

The robot benefits Dahlia’s children and not Dahlia herself. This is a way of throwing the responsibility of taking care of Dahlia onto the robot (P5, a daughter).

#### Sub-theme 2.2 Participants’ lack of technological literacy

Participants generally expressed a lack of knowledge about technologies that could be used in the care of older people, and most had not heard that robots could be used in any way in this context. The present study marked the first time they had been exposed to the topic.

This is the first time I’ve heard that robots are being developed to care for the elderly. I have no information on this subject (P17, a son).

I’ve heard of robot waiters, that work in restaurants. But I didn’t know there were robots that helped the elderly (P15, a son).

This lack of literacy was not limited to robots. Family caregivers had little knowledge as well about other technologies that could be used in the care of older people. For instance, although participants reported positive attitudes toward the use of non-invasive monitoring technologies (e.g., sensors) as a supporting tool in the care of older people, they did not have sufficient knowledge about such technologies, nor did they know how or where to obtain them.

I’m in favour of using sensors, good technologies. I don’t use them because I haven’t even heard that sensors can be used in the care of the elderly. I heard that sensors can be used so that if you forget your baby in the car, for example, it will alert you. That’s what I heard (P5, a daughter).

I wanted to use drop sensors, but they were hard to come by (P1, a daughter).

#### Sub-theme 2.3 Occurrence of system malfunctions

Participants reported that it was impossible to rely on robots because of possible malfunctions that could occur in the system. Using a robot could therefore endanger the lives of older people. Along these same lines, robots might not know how to correctly or adequately meet the needs of older people.

I don’t think that a robot would increase her [Dahlia’s] independence. Rather, it could endanger her life […] a malfunction could occur in the system and then could, for example, provide medication incorrectly (P9, a daughter).

Dahlia may ask the robot for water, and then suddenly it stops working due to a malfunction, or it gets confused in remembering the medicines, and then the woman [Dahlia] dies because of it (P10, a daughter).

#### Sub-theme 2.4 Loneliness

Participants indicated that using robots to assist with elder care might increase the risk of the older person’s loneliness. If family members begin to rely more on robots in the care of their loved one, then their visits will naturally decrease:

[...] because the children can rely on the robot, they will slowly forget her, and visit her less (P16, a daughter).

Dahlia could end up feeling lonely. Usually there is a TV, there is a radio, those things help in terms of entertainment. But the robot has an even more difficult role. It is not a human being and so she [Dahlia] won’t feel there is another person in her life [...] It is likely that her children will depend on the robot, and then Dahlia will become a secondary and not a central thing in their lives (P10, a daughter).

The participants stated that the most important thing for older people was to be surrounded by their children and grandchildren, and not to be alone. Therefore, as mentioned earlier, the family caregivers emphasized that regardless of the task, they were opposed to the robot’s assistance. They believed that older people expected help not only with housework (e.g., cleaning, laundry) or personal care (e.g., bathing). They also expected to have social interactions during the performance of those functional tasks (e.g., talking, exchanging news, receiving updates on family members and acquaintances).

I also don’t agree to the use of a robot for even a few hours to help Mom with the housework, because it’s not a matter of housework... When they become old, people feel lonely.... When I come to Mom to clean the house and then leave, she gets sad, because she wants to talk to me, to talk about life and what’s been happening with her, with my children, and with the neighbours (P7, a daughter).

Yes, I think it [using a robot] is not right [...] it’s impossible to do that. A worker should be brought in to help her if the children cannot do so. Life is not just about cleaning the house; it is easy to wash, the matter is not a matter of washing. She wants someone to talk to her about what is going on in her life (P3, a daughter).

## Discussion

The purpose of the present study was to expand our understanding of the social and ethical issues pertaining to the use of robots in the care of older people, among a culture that has not previously been investigated in this context. Specifically, in exploring the attitudes of Arab family caregivers in Israel (i.e., individuals who come from a collectivist, traditional, Middle-Eastern culture), we found that compared to studies conducted in East Asian or Western cultures (Mast et al. 2010; Kodate et al. 2022; Suwa et al. 2020; Yuan et al. 2022; Wang et al. 2017), the majority of our participants had negative attitudes toward robot-assisted care for older people. They were strongly opposed to adopting such care for their loved ones, regardless of the time required for the robot-conducted task at hand.

A novel finding of the current study was the filial/moral obligation to provide care for older people as a reason not to use robots in elder care. Indeed, Arab society in Israel is characterized by a high commitment to caring for older parents, and using a robot for such care implies the abandonment of parents and the handing over of their care to non-living entities (i.e., robots), a phenomenon which does not align with family values. Note that the obligation to care for one’s parents is part of Islam and is mentioned in the Quran (Bensaid and Grine 2014; Setyawati et al. 2024). In Arab society in Israel, as well as other Arab societies, Islam is an integral part of culture, shaping moral and cultural values as well as norms of acceptable behaviour (Al-Kandari and Gaither 2011). In addition, several Hadiths (i.e., a form of Islamic oral tradition containing the purported words, actions, and silent approvals of the prophet Muhammad), such as the prophet Muhammad’s saying, “Paradise lies at the feet of mothers” (Sunan al-Nasa’i 3104), fatwas, and ijtihad rulings issued by religious leaders across the Islamic world highlight the moral and religious obligation of filial care, reinforcing its importance in the Muslim community. At the same time, studies have indicated that the familial obligation to care for older family members may have negative consequences for caregivers: they may take on the responsibility of caring for their parents even though doing so creates conflicts at work or in the family, thus increasing the burden of care, symptoms of depression, and the arousal of ambivalent feelings toward the caring of their loved ones (AboJabel et al. 2021; AboJabel and Werner 2022).

Another interesting finding of the study was participants’ emphasis on the option of using formal services in the event that family members themselves could not provide the required care to their older family members. Arab society in Israel is known to consume fewer formal services than the majority population (i.e., Jews). This is due partly to cultural beliefs, for example, the stigma associated with receiving social services from the state involves negative emotional reactions among individuals such as shame (Werner and Tur-Sinai 2024). The underconsumption of services is also due to lack of formal services in general for the Arab population since they often live in remote areas. Finally, there is lack of services that are culturally adapted for older Arabs, who often need to overcome language barriers due to poor command of the Hebrew language (Chernichovsky et al. 2017a, b; Naim et al. 2017; Shibli et al. 2021; Vitman-Schorr and Khalaila 2022; Werner and Tur-Sinai 2024). These findings suggest that despite what we have described above, family caregivers still prefer to use formal services such as nursing home and employing caregivers because at least the human contact still exists.

Another reason that participants expressed a preference for humans over robots in the care of older people was the risk of endangering them as a result of possible system failures. Concerns about technology malfunctions, safety, and reliability have previously been raised in studies conducted in Germany, the United States, and Australia, and also among Israeli Jews, not only among family members, but also among older people, the general public, and professionals who take care of old people (AboJabel et al. 2024; AboJabel and Ayalon 2023; Maan and Gunawardana 2017; Mitzner et al. 2010; Müller et al. 2017; Papadopoulos et al. 2020; Tsertsidis et al. 2019). The roots of such concerns lie in the fact that these technologies are still in their infancy (Honig and Oron-Gilad 2018). Since human safety and avoiding physical or intellectual danger is one of the cornerstones of designing robots, many studies focus on improving the reliability and safety of robots (Martínez-López, et al. 2023). However, it is possible that the doubts raised by the participants in the current study are related to lack of familiarity with robots and to lack of technological literacy. Indeed, most of the participants did not know that there were robots designed to help care for old people, and in fact this lack of literacy was not specific to robots but to the use of technology for elderly care in general. Studies have shown that lack of technological literacy is related to negative attitudes toward technology (Avsec and Jamšek 2018). Therefore, in order to promote the use of technology in general in the care of older people, technology literacy levels must first increase. In this context, welfare and health services may play an important role in promoting technological literacy by increasing knowledge and exposure of family caregivers to technology and to robots in particular, focusing on how they can meet the specific needs of older people and their family members.

Finally, risk of loneliness as a result of robot-assisted care was another factor stressed by the study participants as a reason not to use robots for elder care. That said, the research on robots and loneliness has not yielded unequivocal results. Some studies have indeed shown that robots such as those that help with housework (i.e., domestic robots) or robots that facilitate remote communication may reduce human interaction and companionship by reducing the number of family member visits, or allowing these visits to take place virtually, thus increasing the risk of loneliness (Rantanen et al. 2018; Sharkey and Sharkey 2012). Other studies, however, have shown that companion robots or robots that facilitate communication between the older person and the environment may actually reduce loneliness (Gasteiger 2021; Pirhonen et al. 2020). Given that loneliness is a problem for older people worldwide and is associated with devastating physical and psychological health consequences (Courtin and Knapp 2017; Park et al. 2020), scholars must be mindful of these issues and conduct sophisticated studies so as to unveil the reality of the situation.

## Limitations and Conclusions of the Study

The present study had some limitations. First, in terms of the demographic characteristics of the participants, given the explorative nature of this study, we decided that a homogenous population would be best, and therefore the study comprised only Muslim family caregivers; that is, we did not include family caregivers from other religions such as Christians or Druze. We intend to expand our research to other populations in future studies. Further, we investigated the attitudes of adult children of older people; we did not investigate the attitudes of other family caregivers, such as spouses. Therefore, the findings of the study do not reflect the attitudes of all Arab family caregivers in Israel. In addition, most of the participants were women. Women traditionally take on the responsibility of care more than do men, and are willing to spend more time providing emotional and instrumental care (Bartlett et al. 2018). However, this aspect could have affected the results of the study, as men generally have more positive attitudes to technology (Cai et al. 2017; Goswami and Dutta 2015). Furthermore, we did not consider whether the participants in the current study were employed or not. Providing care to family members alongside holding down a job may create many unique challenges among family members who are caregivers, for example conflicts between work demands and their role as caregivers, high level of stress, caregiving burden, and poor mental health such as depression (Boumans and Dorant 2021; O’Neill et al. 2022). While technology may provide a solution to these challenges (Spann et al. 2020), it can be assumed that employment status may influence the attitudes of family members to robots as helping in the care of older people. Future studies should address these aspects. Second, in the present study we examined only the attitudes of family caregivers, not the attitudes of the older persons themselves, or those of other stakeholders, such as professionals or the public. Future studies will have to examine these groups to provide a more comprehensive view of the potential willingness to adopt robots and recommendations as to how to promote their use. Third, data collection was conducted face-to-face or via telephone interviews. As a result, we cannot rule out the effect of social acceptability, as participants may express attitudes that are consistent with accepted social norms (Karatsareas 2022). Relatedly, the way the vignette was presented by the researchers may have influenced participants’ perceptions and attitudes. Finally, although financial concerns related to purchasing robots were not detected in the current study, it would be reasonable that this limitation exists, so future studies should address this issue.

Despite these limitations, the present study has important implications. Filial/moral obligation to take care of one’s elders emerged for the first time as a factor leading family members to choose human over robot care. Along with this, they identified other factors that could constitute barriers to the use of robots, including a lack of technological literacy and concerns with system malfunctions, as well as the increased risk of loneliness. The growing need to care for old people, due to their increasing numbers, and the challenges and burden of care that can accompany the aging of the population may be addressed by robots, as studies show that they have a high potential to be effective for the older person and for the family members (Kim et al. 2022; Samuel et al. 2023; Shishehgar et al. 2018; Suzumura et al. 2020). Therefore, it is necessary to develop intervention programmes among family caregivers aimed at increasing technological literacy and which clarify the advantages and disadvantages of using robots and how to use technology and robots without risk, thus reducing negative attitudes (Johansson-Pajala et al. 2019). Finally, such interventions must be “culturally competent” (Anderson et al. 2003), so that family caregivers do not feel a conflict between their cultural values and the use of robots in elder care. Such a conflict would only lead to an increase in negative attitudes toward technology and ultimately their rejection.

## Data Availability

The data that support the findings of this study are available from the corresponding author upon reasonable request.

## Appendix 1

**Opening Questions**

1. Do you like technology? What technology do you use in your daily life? For what purposes?
2. Do you use any technology to care for your relative? Which one/ones? For what purposes?
3. Have you heard about/been exposed to the topic of robots (in your life, on social media, on TV)? What do you know about robots?
4. What comes to mind/what do you feel when you hear the word robot?
5. Are you familiar with/have you been exposed to the idea that there are robots to care for older people? What do you know about robots in this context?

**Key Questions:**

Now I want to read you a hypothetical story, and then I would like to hear your opinion on a number of issues related to this story.

Dahlia is seventy years old, a widow with three children who lives alone in a large apartment. Dahlia has high blood pressure and diabetes. A few months ago, Dahlia fell at home, and since then she has difficulty moving in the house (e.g., moving from the bed to a chair), performing daily activities (e.g., taking a shower), and household chores (e.g., cleaning or cooking). In order to help, her children decided to buy a robot. Dahlia can communicate with the robot by voice, and she can ask the robot to lift her onto the bed or a chair, bring her water or food from the refrigerator, put the dishes in the dishwasher, turn on the washing machine, and open the door. In addition, the robot can remind Dahlia to take her medication, and offer her various types of activities for pleasure, such as listening to music or watching movies. Also, the children can communicate with Dahlia through the robot and ask how she is. They can talk to her and see her through a camera installed in it.

1. How might using a robot affect Dahlia (e.g., promote her independence, endanger her, etc.)?
2. How might using a robot affect Dahlia’s children?
3. What do you think of the way the children care for Dahlia? Would you suggest another way?
4. Would you be ready to use a robot in the care of your relative? For several hours? To perform one or several functions mentioned in the story (e.g., communication, medication reminder)? Why?
5. How could a robot help you deal with the challenges related to caring for your loved one?
6. Would you be willing to use other technologies (besides a robot) in the care of your loved one, such as cameras, sensors (e.g., fall detectors), GPS watches, panic buttons, etc.?

**Closing Question:**

Thank you very much for your cooperation. Would you like to add anything else that we did not discuss during the interview?
